# Study of Correlation of Severity of Diabetic Retinopathy with Corneal Thickness and Endothelial Parameter Changes in Diabetic Patients

**DOI:** 10.22336/rjo.2025.87

**Published:** 2025

**Authors:** Simran Dhami, Avadhesh Oli

**Affiliations:** 1 Vitreo-retina and Uvea division, Department of Ophthalmology, Command Hospital (Air Force), Bangalore, India

**Keywords:** corneal endothelium, specular microscopy, hexagonality, diabetic retinopathy, diabetic keratopathy, AGEs = Advanced glycation end products, CCT = Central corneal thickness, CV = coefficient of variation, DM = Diabetes mellitus, DR = Diabetic retinopathy, ECD = Endothelial cell density, HbA1c = glycated haemoglobin, HEX = Hexagonality of endothelial cells, IOP = Intra-ocular pressure

## Abstract

**Background and Objective:**

Corneal endothelium plays an essential role in maintaining corneal transparency and hydration. Conditions or events causing endothelial cell loss beyond the threshold value can lead to loss of corneal transparency. Our study aimed to assess the effects of duration of Type 2 Diabetes Mellitus and diabetic retinopathy (DR) severity on the corneal endothelium using non-contact specular microscopy.

**Materials and methods:**

A prospective study was conducted on 200 eyes of diabetic patients and their age-matched controls. On their first visit, a detailed history including diabetic status, duration of diabetes, and previous eye surgeries was taken. A comprehensive ophthalmic evaluation was done. Corneal endothelial parameters like Central Corneal Thickness (CCT), Endothelial Cell Density (ECD), Coefficient of Variation (CV), and hexagonality were obtained using a non-contact Specular microscope. Data was tabulated and analysed using appropriate statistical tests, and a “p” value of less than 0.05 was considered statistically significant.

**Results:**

Endothelial cell hexagonality and duration of diabetes were found to be significantly correlated (p value of 0.001 using an independent t-test), indicating that a longer duration of diabetes causes morphological changes in the corneal endothelium. Other parameters, such as CCT, CV, and ECD, did not correlate significantly with HbA1c levels, diabetes duration, or DR severity.

**Discussion:**

In our study, we evaluated all endothelial parameters and correlated them with the duration of DM (<10 yrs and >10 yrs). Average ECD, CV, and HEX in the two subgroups were 2494.3 ± 405.2 cells/mm^2^ and 2473.1 ± 321.1 cells/mm^2^, 39.1 ± 4.8% and 37.8 ± 4.6%, and 45.6 ± 6.5% and 43 ± 6%, respectively. A significant positive correlation (p value of 0.00) was found between the duration of DM and HEX; however, no significant correlation was found between the duration of DM and ECD (p value of 0.774) and CV (p value of 0.154). Decreased HEX with increasing DM duration suggested that, over time, diabetes induces morphological changes that may eventually compromise endothelial health.

**Interpretation and Conclusion:**

Our findings indicate that corneal endothelial cells undergo morphological changes with increasing duration of Diabetes. The findings of this study add to the literature on the effects of Diabetes on endothelial health, which might help improve the management of these patients. Future longitudinal studies with larger, diverse cohorts are recommended to validate these associations and explore therapeutic interventions to preserve corneal health in diabetes.

## Introduction

Diabetes Mellitus (DM) is a chronic metabolic disorder and has become a global health challenge, with its prevalence rising steadily across both developed and developing nations. According to 2019 data from the International Diabetes Federation, globally, an estimated 537 million adults are living with diabetes [**1**]. The prevalence of diabetes is increasing rapidly; 2019 estimates put the number at 463 million people living with diabetes [**2**]. The number is projected to reach 643 million by 2030 [**1**], or 7,079 individuals per 100,000, with a continued rise across all regions of the world [**2**]. Diabetes affects all the layers of the eye. It can cause several serious eye complications, which are rapidly becoming the leading cause of ocular morbidity, but can be avoided if they are detected and treated early. Emerging evidence has highlighted that diabetes affects the corneal endothelium and central corneal thickness (CCT), often without overt clinical symptoms until advanced stages.

Endothelial damage is likely due to non-enzymatic glycosylation of proteins and abnormal sorbitol accumulation caused by chronic hyperglycaemia. Accumulation of Advanced Glycation End products (AGEs) disturbs the endothelial cell metabolism and causes a decrease in corneal endothelial cells with aging [**3**]. Other possible mechanisms include mitochondrial dysfunction, which results in the accumulation of Reactive Oxygen Species and mitochondrial injury [**4,5**].

When planning a surgical procedure, such as cataract surgery, corneal health evaluation takes precedence. In patients with diabetic keratopathy, a thorough preoperative evaluation aids surgical planning, reduces postoperative complications, and improves visual outcomes. There is currently no clear consensus from the numerous studies on the topic regarding the connection between endothelial cell alterations and the severity of DR. The severity of the DR may not directly impact the integrity of the corneal endothelium. Our study aims to close the evidence gap regarding the possible relationship between alterations in corneal endothelial cells and the severity of diabetic retinopathy.

In this study, we examined changes in endothelial cells and central corneal thickness in patients with type 2 DM and their age-matched controls. Glycosylated haemoglobin levels, duration, and the severity of diabetic retinopathy were also examined in relation to CCT and various endothelial cell parameters. Primary objectives of the study were to analyse the central corneal thickness and endothelial cell changes like endothelial cell density and morphology in patients with diabetes mellitus, to assess CCT and endothelial cell changes including endothelial cell density (ECD), coefficient of variation (CV), and hexagonality (HEX) using specular microscopy in patients with type 2 DM and compare it with their age matched controls. The influence of diabetic retinopathy severity, DM duration, and glycosylated haemoglobin levels on CCT and endothelial cell changes was also analysed.

## Materials and methods

**Fig. 1:**
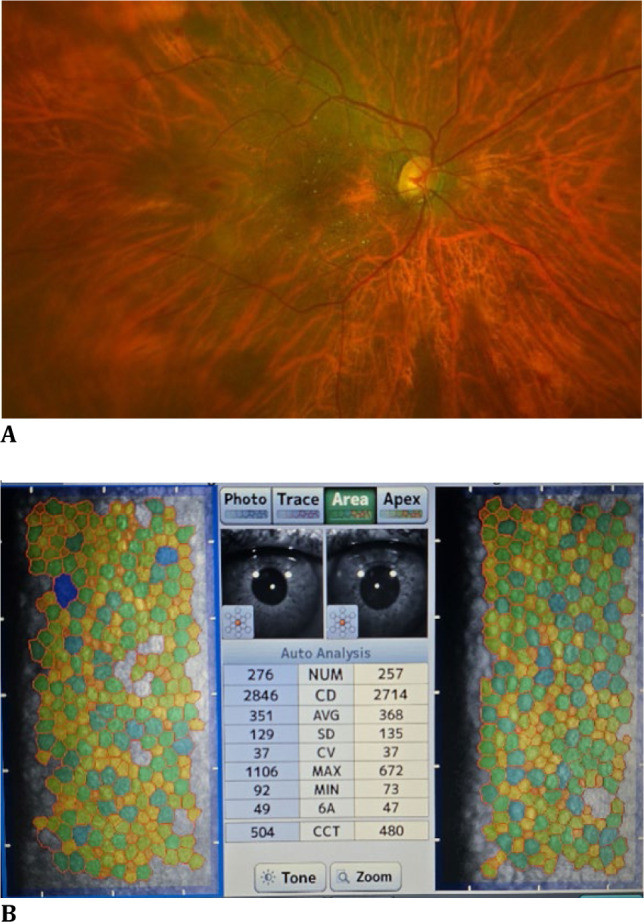
**A, B** Wide-field fundus and specular microscopic images of the right eye of a 52-year-old female patient with moderate non-proliferative diabetic retinopathy

This prospective study was conducted in the OPD of the Department of Ophthalmology in a tertiary care hospital. Approval and clearance from the institutional ethics committee were obtained, and patients who met the inclusion criteria were enrolled in the study after providing informed consent. All diabetic patients were enrolled as cases, and their relatives or other non-diabetic patients with minor ocular ailments were enrolled as controls. General, ophthalmic, and systemic disease history was obtained from all participants. Information about age, gender, current occupation, presence of concomitant systemic diseases, topical and systemic medications, and any history of ocular surgery was obtained. Details on the current HbA1c levels of diabetic patients were obtained, and patients were divided into three subgroups based on HbA1c levels (<6.5%, 6.5%–7.5%, and >7.5%). Based on duration, diabetic patients were further subdivided into two subgroups (<10 years and >10 years). A complete ocular examination was performed for all diabetic patients with diabetic retinopathy and their age-matched controls. Vision was recorded using digital LogMar charts in LogMar equivalents. IOP was recorded using a TOPCON non-contact tonometer. A detailed fundus examination was performed using indirect ophthalmoscopy with a 20D lens and the Nikon OPTOS Ultra-widefield retinal imaging system. Assessment of corneal endothelial thickness and morphology was performed for all patients on their first visit using the TOMEY EM-4000 specular microscope (**Fig. 1A, B**).

All participants in this study were evaluated in a sitting position using the Tomey 4000 non-contact specular microscope, and an average of 3 readings was obtained for each eye.

Findings from specular microscopy were clinically correlated with slit lamp examination. Specular microscopy was used to assess CCT, ECD, CV, and HEX.

### Inclusion criteria

All patients presenting to the ophthalmology department with diabetes, with or without diabetic retinopathy, and their age-matched non-diabetic controls (50-70 years) who were willing to participate in the study.

### Exclusion criteria

All conditions affecting or altering the health of the corneal endothelium were excluded from the study. These conditions included corneal diseases such as corneal endothelial dystrophies and Fuchs endothelial dystrophy; prior ophthalmic surgeries; severe ocular trauma; glaucoma; previous retinal photocoagulation or intravitreal injection; present or past uveitis; and ocular infections.

### Statistical analysis

Descriptive statistics for the explanatory and outcome variables were calculated as means and standard deviations for quantitative variables, and as frequencies and proportions for qualitative variables. Inferential statistics, such as the Chi-square test, were applied to qualitative variables to assess association; the independent-samples t-test was applied to compare quantitative parameters between groups; and the ANOVA test was applied to compare mean quantitative parameters among groups. Pearson’s correlation was used to assess the association between endothelial parameters and HbA1C and DM duration. The level of significance was set at 5%. Statistical analysis was performed using the SPSS (Statistical Package for Social Sciences) version 21 (IBM SPSS Statistics - IBM corporation: NY, USA).

## Results

This study included 200 eyes from 200 patients: 100 cases (diabetic patients) and 100 non-diabetic age-matched controls. Patients aged 50-70 years were included in the study, with a mean age of 61.22 (SD ± 5.55) years, and controls, 60.12 (SD ± 5.28) years; the two groups had similar age distributions (p=0.152). Of the 200 patients, the diabetic group has 59% males and 41% females, whereas the diabetic group has 46% males and 54% females. The diabetic group had more males than the non-diabetic group (46%); however, the slight gender imbalance was not statistically significant (p=0.066). HbA1c levels amongst the people with diabetes were found to be significantly higher compared to non-diabetics (7.79 vs. 5.66, p=0.001), confirming poor glycaemic control among the diabetic patients. No significant differences in visual acuity (BCVA) or intraocular pressure (IOP) were noted between the two groups (p>0.05). Hypertension (46%-59%) and hypothyroidism (47%-59%) were amongst the leading associated co-morbidities found in both groups. **Table 1** shows a detailed demographic distribution of the study. **Table 2** presents the diabetes parameters included in the study and their distribution. 91% of diabetics had HbA1C ≥ 6.5 (50% in 6.5–7.5 range; 41% >7.5). 64% of cases showed some degree of DR (24% moderate, 19% severe/PDR). Approximately 47% diabetic cases have had a duration of ≥ 10 years (5% > 20 years).

**Table 1:** Demographic data of all the 200 cases and their age-matched controls

Parameter	Diabetic Group (mean ± SD)	Non-Diabetic Group (mean ± SD)
**Age (years)**	61.22 ± 5.55	60.12 ± 5.28
**Sex**	Males: 59% Females: 41%	Males: 46% Females: 54%
**HbA1c Levels (%) ± SD**	7.79 ± 1.46	5.66 ± 0.59
**BCVA (LogMar Equivalent)**	0.36 ± 0.53	0.45 ± 0.62
**IOP (mmHg)**	18.04 ± 2.78	16.94 ± 2.67
**Co-morbidities** **1. Hypertension (HTN)**	59 %	46%
**2. Coronary Artery Disease (CAD)**	8%	7%
**3. Chronic Obstructive**	3%	3%
**4. Pulmonary Disease (COPD)**	2%	3%
**5. Chronic Kidney Disease (CKD)**	2%	2%
**6. Anaemia**	6%	8%
**7. Hypothyroidism**	59%	47%

BCVA = best corrected visual acuity, IOP = intraocular pressure, HbA1c = glycated haemoglobin

**Table 2:** Distribution of study data according to HbA1c levels, severity of diabetes, and duration of diabetes amongst the diabetic group

**HbA1c Levels**	< 6.5: 9%
	6.5–7.5: 50%
	> 7.5: 41%
**Severity of DR**	No DR: 36%
	Mild DR: 21%
	Moderate DR: 24%
	Severe DR: 10%
	PDR: 9%
**Duration of DM**	< 10 years: 53%
	10-20 years: 42%
	> 20 years: 5%

Association of CCT and endothelial parameters with HbA1c levels, duration of diabetes, and severity of diabetes is depicted in **Tables 3, 4,** and **5**. No significant differences in CCT and corneal endothelial parameters, including ECD, CV, and HEX, were observed across HbA1C groups (all p>0.05) using ANOVA, indicating that the level of glycaemic control (HbA1C) did not directly affect endothelial health. A significant increase in HEX was observed in diabetic patients with more than 10 years of diabetes duration (47.6 vs. 42.3, p=0.001) using an independent-samples t-test, suggesting that longer diabetes duration may alter endothelial cell shape (hexagonality). A near-significant increase in CCT was observed in the > 10-year group (511.7 vs. 501.3 μm, p=0.163), suggesting that prolonged diabetes may affect corneal thickness. No significant differences were observed in ECD, CV, HEX, or CCT across all grades of DR (all p>0.05). However, severe DR showed numerically higher ECD (2635.7 cells/mm^2^), but this difference was not statistically significant. This suggests that the severity of diabetic retinopathy does not directly correlate with endothelial damage.

**Table 3:** Association of HbA1c Levels with Endothelial Parameters and CCT

Parameter	< 6.5%	6.5-7.5%	> 7.5%	p-value
**ECD (cells/mm^2^)**	2490.0 (2078-2751)	2460.9 (1371-3003)	2511.6 (1215-3349)	0.808
**CV (%)**	37.6 (31.0-51.0)	38.1 (31.0-50.0)	39.1 (31.0-51.0)	0.517
**HEX (%)**	45.7 (31.0-53.0)	43.8 (31.0-55.0)	44.5 (30.0-56.0)	0.697
**CCT (μm)**	490.1 (437.0-524.0)	504.4 (398.0-585.0)	511.9 (467.0-592.0)	0.256

**Table 4:** Association of Duration of DM with Endothelial Parameters and CCT

Parameter	< 10 years	> 10 years	p-value
**ECD (cells/mm^2^)**	2473.1 (1215-2933)	2494.3 (1371-3349)	0.774
**CV (%)**	37.8 (31.0-51.0)	39.1 (31.0-51.0)	0.154
**HEX (%)**	47.6 (30.0-55.0)	42.3 (35.0-58.0)	0.00*
**CCT (μm)**	511.7 (446.0-587.0)	501.3 (398.0-592.0)	0.163

**Table 5:** Association of Severity of DR with Endothelial Parameters and C

Parameter	No DR	Mild NPDR	Moderate NPDR	Severe NPDR	PDR	p-value
**ECD (cells/mm^2^)**	2452.4 (1371-3003)	2476.1 (1386-2908)	2451.7 (1215-2934)	2635.7 (1992-3349)	2549.8 (2214-2933)	0.658
**CV (%)**	38.1 (31.0-50.0)	38.9 (31.0-51.0)	39.4 (32.0-51.0)	38.6 (32.0-50.0)	36.8 (31.0-42.0)	0.661
**HEX (%)**	43.5 (31.0-54.0)	45.6 (31.0-54.0)	43.3 (33.0-55.0)	44.3 (30.0-56.0)	46.7 (40.0-52.0)	0.510
**CCT (μm)**	493.5 (398.0-585.0)	512.3 (454.0-569.0)	515.1 (465.0-572.0)	514.0 (471.0-592.0)	510.1 (478.0-587.0)	0.154

Using Pearson’s analysis, a positive correlation with HEX (r=0.205, p=0.00) was noted with the increasing duration of diabetes. No significant correlation was found between endothelial parameters and CCT or HbA1c levels (**Table 6**). Using an independent-samples t-test to compare endothelial parameters and CCT between the two groups, people with diabetes had higher CCT (506.19 vs. 496.66 μm; p = 0.049), which was significant. No differences between the groups were noted regarding endothelial parameters (**Table 7**).

**Table 6:** Correlation of Corneal Changes with Duration, HbA1C, and DR Status

Factor	ECD	CV	HEX	CCT
**Duration of DM** **r value** **p value**	-0.029 0.774	-0.144 0.154	0.205 0.00*	0.141 0.163
**HbA1C** **r value** **p value**	-0.016 0.874	0.057 0.575	-0.031 0.757	0.180 0.073

**Table 7:** Comparison of CCT and endothelial parameters amongst cases and controls

Parameter	Diabetic Group	Non-Diabetic Group	p-value
**ECD (cells/mm^2^)**	2484.30 (1215-3349)	2479.39 (1351-3154)	0.919
**CV (%)**	38.49 (31.0-51.0)	38.09 (26.0-66.0)	0.593
**HEX (%)**	44.26 (30.0-56.0)	44.93 (27.0-64.0)	0.504
**CCT (** **μm)**	506.19 (398.0-592.0)	496.66 (428.0-559.0)	0.049*

## Discussion

Our study found a significant increase in CCT (p value of 0.049) in diabetic cases (506.19 ± 37.22 μm) compared with their age-matched controls (496.66 ± 30.56 μm). In 2023, a case-control study found that the mean CCT was higher in diabetic patients than in non-diabetic controls; however, this difference was not statistically significant. Notably, a substantial increase in CCT was observed with advancing grades of diabetic retinopathy (DR), and they concluded that overall CCT differences between diabetics and non-diabetics were minimal. Still, CCT increased significantly with DR severity [**6**]. A study by Reddy CV et al. found a significant difference (p<0.0001) in CCT between diabetic corneas and non-diabetic controls. Additionally, CCT was found to be higher in people with diabetes with a disease duration of over 10 years, compared to those with a shorter duration [**7**]. Elevated HbA1c levels (> 6.5%) were also found to be associated with increased CCT. ​They concluded that diabetic individuals have thicker corneas, with CCT increasing alongside disease duration and poorer glycaemic control. In 2024, a study of 124 diabetic patients found a significant difference in CCT compared with non-diabetic individuals [**8**]. Another study found concurrence with the previous research. It showed that CCT was higher in people with diabetes with a disease duration of over 10 years compared to those with a shorter duration [**9**]. Additionally, they found that elevated HbA1c levels (> 6.5%) were associated with increased CCT. In summary, these studies collectively indicate that individuals with diabetes tend to have increased central corneal thickness compared to non-diabetic individuals, and our study found concurrence with previous studies. These findings underscore the importance of regular corneal assessments in diabetic patients to monitor and manage potential ocular complications effectively.

In our study, we divided the duration of diabetes into two groups: less than 10 years and more than 10 years. In the < 10 years group, the average CCT was 501.3 ± 42 μm, while in the > 10 years group it was 511.7 ± 30.4 μm; no significant correlation (p value = 0.163) was found between diabetes duration and CCT. Previous studies on the same have found conflicting results. In 2017, one study found a positive correlation between thicker corneas and longer diabetes duration [**10**].

In our study, we divided DR severity into four grades (no DR, mild DR, moderate DR, severe DR, PDR). Average CCT in no, mild, moderate, severe DR, and PDR was found to be 493.5 ± 43 μm, 512.3 ± 32 μm, 515.3 ± 29.6 μm, 514 ± 38.4 μm, and 510.1 ± 34 μm, respectively. No significant correlation (p-value = 0.154) was observed between DR severity and CCT. Previous literature has provided varied results in this regard. In 2024, while finding out the correlation of CCT and keratometry findings with the severity of DR and glycosylated haemoglobin (HbA1c) levels in Type II diabetes mellitus patients, a study suggested that individuals with DR generally have a higher CCT compared to the control group, particularly in the earlier stages of the disease (no DR and mild non-proliferative diabetic retinopathy). However, as the severity of DR increases, the CCT tends to decrease slightly in moderate and severe stages [**11**]. However, a 2020 study found no significant difference in CCT between patients with and without DR. Additionally, no correlation was found between CCT and disease duration, retinopathy severity, or prior laser therapy in their study [**12**].

In our study, we divided HbA1c levels into three groups (<6.5%, 6.5-7.5%, and >7.5%) and found average CCTs of 490.1 ± 30.5 μm, 504.4 ± 42.4 μm, and 511.9 ± 30.8 μm, respectively. No significant correlation (p value of 0.256) was noted between the HbA1c level and CCT. Previous literature found significantly higher CCT in diabetic patients, suggesting an association between increased CCT and hyperglycaemia [**13**]. It was also found that diabetes affects corneal endothelial cells in older individuals and those with long-standing diabetes and higher HbA1c levels, implying a relationship between elevated HbA1c and corneal changes [**7**].

A cross-sectional study published in 2024 contradicts previous studies and found no statistically significant relationship between CCT and HbA1c levels in Type II diabetes mellitus patients, suggesting that glycaemic control may not directly influence corneal thickness [**11**].

In our study, we evaluated all endothelial parameters by dividing the duration of DM into two subgroups: less than 10 years and more than 10 years. Average ECD, CV, and HEX in the two subgroups were 2494.3 ± 405.2 cells/mm^2^ and 2473.1 ± 321.1 cells/mm^2^, 39.1 ± 4.8% and 37.8 ± 4.6%, and 45.6 ± 6.5% and 43 ± 6%, respectively. No significant correlations were found between the duration of DM and ECD (p value of 0.774) or CV (p value of 0.154); however, a significant positive correlation (p value of 0.00) was found between the duration of DM and HEX. HEX decreased with increasing DM duration, suggesting that, over time, diabetes induces morphological changes that may eventually compromise endothelial health. In 2020, a study by Çolak S et al. concluded that longer duration of diabetes was associated with decreased ECD and increased CV, indicating greater variability in cell size. However, no significant difference in hexagonality was observed between diabetic patients and healthy controls [**14**]. Another 2022 study demonstrated a significant positive correlation between CV and diabetes duration, suggesting that longer disease duration is associated with greater cell size variability. However, no significant association was found between ECD or hexagonality and diabetes duration in the study [**15**]. These studies collectively suggest that longer durations of diabetes are associated with decreased ECD and increased CV, reflecting greater variability in cell size. The impact on hexagonality appears to vary among studies, indicating that while some morphological changes are consistently observed with prolonged diabetes, others may be influenced by additional factors.

In our study, we found the average ECD in no, mild, moderate, severe DR, and PDR to be 2452.4 ± 389.1 cells/mm^2^, 2476.1 ± 378.4 cells/mm^2^, 2451.7 ± 368 cells/mm^2^, 2651.7 ± 380.2 cells/mm^2^, and 2549.8 ± 217.4 cells/mm^2^, respectively. No significant correlation (p-value = 0.658) was observed between DR severity and ECD. The average CV was 38.1 ± 4.4%, 38.9 ± 5%, 39.5 ± 5.2%, 38.6 ± 5.6%, and 36.8 ± 3.7% in each subgroup, respectively. No significant correlation (p-value = 0.661) was observed between DR severity and CV. Average HEX was 43.5 ± 5.9%, 45.6 ± 6.5%, 43.3 ± 6.8%, 44.3 ± 8.2%, and 46.7 ± 3.8%, respectively. No significant correlation (p-value = 0.51) was observed between DR severity and HEX.

Several previous studies have examined the association between endothelial parameters and DR severity, but their findings have been inconsistent. A 2019 study reported a significant reduction in ECD and HEX, along with an increase in CV and central corneal thickness, correlating with DR progression [**16**].​ However, a cross-sectional study on 134 eyes of 134 diabetic patients in 2022 found conflicting results and no significant differences in corneal endothelial parameters between diabetic patients with and without DR [**15**]. Another study by Chowdhury B et al. in support of the previous study found no correlation between the severity of DR and pathological alterations in corneal endothelial cells. However, long-term poorly controlled glycemia was noted to affect the corneal endothelium and increase corneal thickness significantly [**17**]. These studies highlight the complex relationship between corneal endothelial parameters and DR severity, suggesting that factors such as glycaemic control and disease duration may play significant roles in corneal endothelial health.

In our study, we found average ECDs of 2490 ± 218.9 cells/mm2, 2460.9 ± 397.1 cells/mm2, and 2511.6 ± 357.8 cells/mm2 in the HbA1c groups, respectively. No significant correlation (p value of 0.808) was noted between the HbA1c levels and ECD. The average CV across the three subgroups was 37.6 ± 6.7%, 38.1 ± 4.3%, and 39.1 ± 4.9%, respectively. No significant correlation (p value of 0.517) was noted between the HbA1c levels and CV. The average HEX in the three subgroups was 43.7 ± 7.2%, 43.8 ± 5.9%, and 44.5 ± 6.8%, respectively. No significant correlation (p value of 0.697) was noted between the HbA1c levels and HEX.

A study by Chowdhury et al. showed that elevated HbA1c levels may contribute to increased corneal endothelial cell size variability [**17**]. Another study showed that higher HbA1c levels are associated with reduced ECD. This suggests that poor glycaemic control may lead to a decrease in the number of corneal endothelial cells [**14**].​ However, a negative correlation between HbA1c levels and HEX was noted in a study, indicating that higher HbA1c levels are associated with a decrease in the percentage of hexagonal cells (pleomorphism). This suggests that poor glycaemic control may alter the shape of corneal endothelial cells. These findings underscore the importance of maintaining optimal glycaemic control to preserve corneal endothelial health in individuals with diabetes mellitus.

In our study, we found the average ECD in the diabetic group to be 2484 ± 366.3 cells/mm^2^, and in the non-diabetic group, 2479 ± 317.4 cells/mm^2^. No significant difference was found between the two groups (p-value = 0.919) using an independent t-test. Other endothelial parameters, including CV (diabetic group 38.49 ± 4.77% and non-diabetic group - 38.09 ± 5.76%) and Hex (diabetic group – 44.26 ± 6.35% and non-diabetic group - 44.93 ± 7.72%), were also not found to be statistically significant using an independent t-test. A cross-sectional study on 120 diabetic patients and 100 controls found that people with diabetes had significantly lower ECD and higher CV, and reduced hexagonal cells in diabetics. They concluded that diabetes is associated with endothelial cell loss, irregular cell size, and changes in cell shape [**10**].

Given previous research and statistical considerations, a well-designed study should include at least 200-300 diabetic patients with varying DR severity (no DR, mild, moderate, severe, and proliferative DR), along with a control group of at least 100-150 non-diabetic controls for comparative analysis. Ideally, each DR subgroup (e.g., mild, moderate, severe, proliferative) should include 50-75 patients to allow meaningful comparisons within subgroups.

### Limitations of this study

Our study had a limited sample size, with only a few patients in different grades of DR. Future longitudinal studies with larger, diverse cohorts are recommended to validate these associations and explore therapeutic interventions to preserve corneal health in diabetes. However, it is recommended that all patients with diabetes undergo a thorough preoperative evaluation before intraocular surgery to improve outcomes.

## Conclusion

Our study of 200 eyes showed that longer diabetes duration significantly affects endothelial cell morphology by increasing hexagonality and polymegathism, which may contribute to corneal dysfunction and ocular complications.

The study underscored the importance of regular endothelial assessments in diabetic patients to facilitate early detection and management of corneal alterations. Additionally, longitudinal studies are needed to better understand disease progression.

Despite these limitations, the insights gained contribute to the existing body of knowledge and emphasize the necessity for further research to refine diagnostic tools and therapeutic strategies. By addressing these gaps, clinicians can enhance patient outcomes and minimize vision-related complications associated with diabetes.

Ultimately, the findings reinforce the need to integrate corneal endothelial evaluation into routine ophthalmic care for individuals with diabetes, thereby enabling better monitoring and management of ocular health in this population. While some of the corneal parameters remained unaffected, these findings underscore the potential of corneal endothelial metrics as non-invasive biomarkers for DR progression, aiding early detection and holistic management. However, a cross-sectional design, a limited sample size, and unmeasured confounders constrain causal inference. Future longitudinal studies with larger, diverse cohorts are crucial to validate these associations, clarify mechanistic pathways, and explore targeted interventions to mitigate corneal endothelial damage, ultimately improving ocular health outcomes in DM.
